# Insomnia among non-depressed multiple sclerosis patients: a cross-sectional study

**DOI:** 10.1186/s41983-018-0016-0

**Published:** 2018-06-15

**Authors:** A. A. Alhazzani, A. Alshahrani, M. Alqahtani, R. Alamri, R. Alqahtani, M. Alqahtani, M. Alahmarii

**Affiliations:** 1 Neurology Section, Department of Medicine, National Guard Health Affairs, King Abdulaziz Medical City, Riyadh, Saudi Arabia; 2 Neurology, Prince Sultan Military Medical City, Riyadh, Saudi Arabia; 3 Neurology, College of Medicine, King Khalid University, Abha, Saudi Arabia; 4 College of Medicine, King Khalid University, PO Box 4557, Abha, 61412 Saudi Arabia; 5 Neurology Section, Department of Medicine, King Khalid University, P.O. Box 641, Abha, Saudi Arabia

**Keywords:** Insomnia, Multiple sclerosis, Depression, Saudi Arabia

## Abstract

**Background:**

Insomnia is a common problem that affects approximately 50% of patients with multiple sclerosis (MS), who suffer from sleep disturbances. In general, persons with insomnia are at a higher risk of developing depression. This study was conducted to assess insomnia among non-depressed MS patients in Saudi Arabia.

**Methods:**

Based on the Patient Health Questionnaire-9 (PHQ-9), those who scored 4 or less for depression out of 598 MS patients were selected (*n* = 112). A cross-sectional study was conducted to interview 112 non-depressed MS patients in order to assess insomnia among them. A data collection sheet has been designed by the researchers. It comprised socio-demographic variables (e.g., gender, age, area of residence, and marital status) and clinical variables (disease duration, age at disease onset, previous diagnosis of depression, and used antidepressant drugs). Insomnia was assessed by the Insomnia Severity Index (ISI), while severity of illness was assessed using the Patient Determined Disease Steps (PDDS)**.**

**Results:**

A total of 72 patients (64.3%) were females, and 62 (55.4%) were married. Their mean age was 32.6 years (SD = 8.9), ranging from 15 to 56 years. As for educational level, 64 (57.1%) had a Bachelor degree. The mean age at disease onset was 26 years (SD = 8.9). The mean duration of illness was 1.9 years. Symptoms of insomnia were present among 14 patients (12.5%). No statistical significance was found between the mean PDSS of insomnia and non-insomnia patients. Significant differences were present between insomnia and non-insomnia patients as regards their education level (*P* = 0.005) and use of antidepressant drugs (*P* = 0.008).

**Conclusions:**

Prevalence of insomnia among non-depressed MS patients is low. Insomnia is associated with educational and use of antidepressants. Further research is needed to assess severity of different types of insomnia among depressed and non-depressed MS patients.

## Background

Multiple sclerosis (MS) is a chronic disease of the central nervous system, with disseminated inflammatory lesions and axonal loss, leading to multifocal signs of neurological deficits (Bamer et al. 2008). Nearly 65% of MS patients suffer from cognitive problems (Rao et al. 1991), resulting in a reduced quality of life (Mitchell et al. 2005). Several factors are thought to negatively influence cognition in MS patients, such as depression (Arnett et al. 2008), fatigue (Krupp and Elkins 2000), and sleep disturbances (Sater et al. 2015).

Approximately 50% of MS patients suffer from sleep disturbances (Bamer et al. 2008). Insomnia, in particular, chronic insomnia, should be treated due to its negative effect on quality of life and functional status (Espie et al. 2016). Obstructive sleep apnea is associated with significant morbidity and mortality and should routinely be sought in MS patients complaining of fatigue and daytime sleepiness (Lunde et al. 2013).

Cross-sectional studies have shown prevalence rates over 40% for insomnia in MS (Stanton et al. 2006; Tachibana et al. 1994), which probably has a multifactorial etiology. Nocturnal symptoms, including cramps, spasms, and neuropathic pain, as well as nocturnal and bladder dysfunction symptoms, are frequent and can contribute to sleep disruption (Merlino et al. 2009). Comorbid psychiatric disorders (such as depression and anxiety) may significantly contribute to insomnia in MS (Pokryszko-Dragan et al. 2013; Lunde et al. 2012).

Up to the best of our knowledge, no study to date examined the relation between depression and insomnia in MS patients in Saudi Arabia. Therefore, this study was conducted to assess insomnia among non-depressed MS patients in Saudi Arabia.

## Methods

The present study followed a cross-sectional design. A medical record review was conducted by the researchers for MS patients registered at tertiary care hospitals in five regions within Saudi Arabia (i.e., southern, east, west, middle, and north). They were diagnosed by a neurologist according to the 2005 revised McDonalds criteria (Polman et al. 2005). MS patients who attained a score of 4 or less for depression based on the Patient Health Questionnaire-9 (PHQ-9) (Kroenke et al., 2001) were included in this study (*n* = 112). All patients were of the relapsing-remitting MS type.

The researchers designed a data collection sheet which was used to interview all 112 selected MS patients. It included a set of socio-demographic variables (gender, age, area of residence, educational level, and marital status) and clinical variables (disease duration, age at disease onset, a previous diagnosis of depression and antidepressant drug use). Severity of MS was measured using the Patient Determined Disease Steps (PDDS), a nine-item patient-administered measure of MS-related disability (Learmonth et al. 2013; Vollmer et al. 1999; Kurtzke 1970; Gulick et al. 2011).

**Fig. 1:**
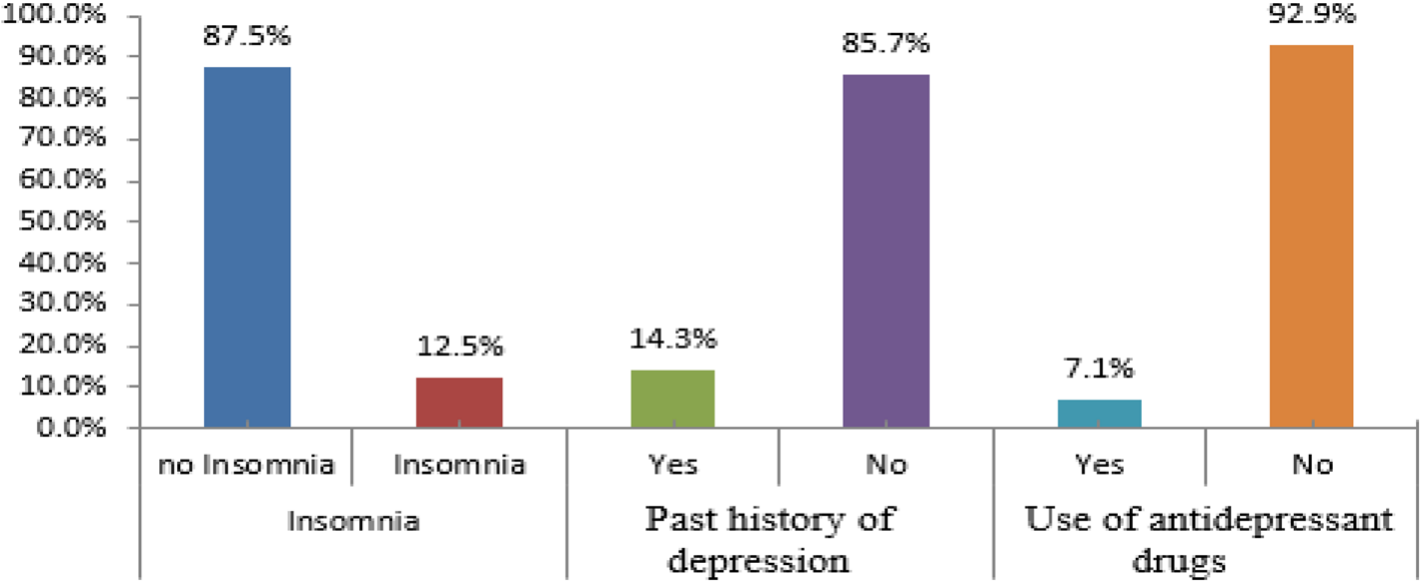
Distribution of non-depressed patients based on the Insomnia Severity Index. The figure shows that 12.5% had insomnia symptoms, 85.7% had past history of depression, and 92.9 and 7.1% used antidepressant drugs

Insomnia was assessed by the Insomnia Severity Index (ISI), a questionnaire widely used to investigate individuals’ potential insomnia and the extent to which insomnia affects their quality of life. It contains seven items that assess an individual’s experience with insomnia on a 5-point Likert scale, with higher scores indicating more severe insomnia (Morin et al. 2011). Based on the ISI, non-depressed MS patients were divided the into two groups, i.e., non-insomnia patients (*n* = 98) with an ISI score of 0–7 and insomnia patients (*n* = 14), who had any degree of insomnia with a score of more than 7, regardless of their type of insomnia (Insomnia Severity Index 2016) (Fig. 1).

The Statistical Package for Social Sciences, (SPSS Inc., Chicago, IL, version 21) was used for the statistical analysis of the data. Descriptive statistics were used to summarize the basic features of the collected data. Group differences were assessed using *t* test to compare the mean independent sample values. When the assumption of the *t* test is not valid, we used the nonparametric Mann–Whitney *U* test. The chi-square test was used to examine the relation between two qualitative variables. In all tests, *P* ≤ 0.05 was considered as statistically significant.

## Results

Table 1 shows that among the 112 non-depressed MS patients, 72 (64.3%) were females, 62 (55.4%) were married while 46 (41.1%) were single. Participant patients’ mean age was 32.6 years (SD = 8.9), ranging from 15 to 56 years. As for educational level, 64 (57.1%) had a Bachelor’s degree and 20 (17.9%) had a secondary degree. A total of 34 (30.4%) patients lived in the southern region (Aseer), 42 (37.5%) in the middle region, 24 (21.4%) in the eastern region, and 12 (10.7%) in the western region. Regarding the clinical characteristics of non-depressed MS patients, the mean age at disease onset was 26 years (SD = 8.2) and the mean duration of illness was 3.5 (SD = 1.9) years. The mean number of admissions was 0.8 (SD = 1.3), while the mean number of attacks during the last 2 years was 1.6 (SD = 1.9). The most commonly received medications were interferon beta-1b (32.1%) and teriflunomide (17.9%). Subcutaneous injection was the main routes for drug administration (46.4%), followed by intramuscular injection (28.6%), orally (17.9%) and intravenously (7.1%).

**Table 1:** Demographic and clinical characteristics of study non-depressed MS patients (*n* = 112)

Characteristics	*N* (%)
Gender	
Male	40 (35.7)
Female	72 (64.3)
Marital status	
Single	46 (41.1)
Married	62 (55.4)
Divorced	4 (3.6)
Education level	
Primary	6 (5.4)
Intermediate	10 (8.9)
Secondary	20 (17.9)
Bachelor	64 (57.1)
Postgraduate	12 (10.7)
Region	
Southern	34 (30.4)
Middle	42 (37.5)
East	24 (21.4)
West	12 (10.7)
Age (years), mean ± SD 32.6 ± 8.9	
Age at disease onset (years), mean ± SD 26 ± 8.2	
Disease duration (years), mean ± SD 3.5 ± 1.9	
Number of admissions, mean ± SD 0.8 ± 1.3	
Number of attacks during last 2 years, mean ± SD 1.6 ± 1.9	
Medications used	
Interferon beta-1b (betaferon)	36 (32.1)
Teriflunomide (aubagio)	20 (17.9)
Dimethylfumarate (tecfidera)	24 (21.4)
Rituximab (rituxan)	18 (16.1)
Alemtuzumab (lemetrada)	14 (12.5)
Routes of drug administration	
Oral	20 (17.9)
Subcutaneous	52 (46.4)
Intramuscular	32 (28.6)
Intravenous	8 (7.1)

Table 2 shows that MS patients’ characteristics (age, age at disease onset, disease duration, number of hospital admissions, number of attacks during the last year and PDDS) did not differ significantly according to having insomnia.

**Table 2:** Comparison of MS patients’ characteristics according to presence of insomnia

	Non-insomnia (*n* = 98)		Insomnia (*n* = 14)		*P* value
Characteristics	Mean	SD	Mean	SD	
Age (years)	32.59	9.09	32.86	7.47	0.917
Age at disease onset (years)	25.76	8.22	27.86	7.81	0.370
Disease duration (years)	8.46	14.95	5.29	3.22	0.711
Number of hospital admissions	0.78	1.38	0.86	1.03	0.299
Number of attacks during last 2 years	1.59	1.87	2.00	2.29	0.606
Patient Determined Disease Steps (PDDS)	1.67	1.33	1.57	1.09	0.965

Table 3 shows that prevalence of insomnia among MS patients differed significantly according to their educational level (*P* = 0.005), being absent among less educated (primary or intermediate levels) patients (0%) and highest among postgraduate educated patients (50%). Moreover, prevalence of insomnia was significantly higher among those who received antidepressant medications (*P* = 0.008). However, prevalence of insomnia did not differ significantly according to patients’ sex, marital status, region, route of medication administration, and past history of depression.

**Table 3:** Associations regarding socio-demographic variables between insomnia and no insomnia groups in non-depressed MS patients

		No insomnia		Insomnia		*P*
Characteristics		No.	%	No.	%	
Sex	Male	32	80.0	8	20.0	0.133
	Female	66	91.7	6	8.3	
Marital status	Single	42	91.3	4	8.7	0.136
	Married	54	87.1	8	12.9	
	Divorced	2	50.0	2	50.0	
Educational level	Primary	6	100.0	0	0.0	0.005
	Intermediate	10	100.0	0	0.0	
	Secondary	18	90.0	2	10.0	
	Bachelor	58	90.6	6	9.4	
	Postgraduate	6	50.0	6	50.0	
Region	Southern	30	88.2	4	11.8	0.525
	Middle	36	85.7	6	14.3	
	East	20	83.3	4	16.7	
	West	12	100.0	0	0.0	
Route of drug administration	Oral	18	90.0	2	10.0	0.861
	SC	48	92.3	4	7.7	
	IM	28	87.5	4	12.5	
	IV	2	100.0	0	0.0	
Past history of depression	Yes	12	75.0	4	25.0	0.114
	No	86	89.6	10	10.4	
Antidepressant medications	Yes	4	50.0	4	50.0	0.008*
	No	94	90.4	10	9.6	

*P value was calculated according to Fisher’s exact test, since more than 25% of expected values were less than 5

## Discussion

To our best knowledge, this is the first study to assess prevalence of insomnia symptoms among non-depressed MS patients in Saudi Arabia. Results demonstrated that only 12.5% of non-depressed MS patients suffered from insomnia. This finding is lower than that reported by Stanton et al. (2006), who found that 42% of MS patients had difficulty in initiating sleep, 53% reported extended awakenings, and 58% reported waking and being unable to return to sleep at least twice per week. However, they did not exclude MS patients with depression.

Moreover, Lunde et al. (2012) reported that depressed MS patients have more sleep disturbance. This indicates that depression may be associated with insomnia. Nevertheless, more studies are needed to assess insomnia among depressed versus non-depressed MS patients.

In another study, Baron et al. (2011) demonstrated that patients with comorbid MS and depression have levels of insomnia symptoms above and beyond those reported of the overall MS population, with over three quarters of the sample reporting clinically significant insomnia symptoms. The study of Gupta and Lahan (2011) found that subjects with depressive disorder showed symptoms of primary insomnia.

Our study revealed no statistically significant differences in prevalence of insomnia among MS patients according to their gender. However, some studies suggested a higher prevalence of sleep disturbances in female patients (Bamer et al. 2008; Lunde et al. 2012).

In this study, the educational level is related to insomnia among non-depressed MS patients, where insomnia increases with a higher level of education.

For our insomnia patients, most were classified as sub-threshold insomnia (ISI score 8–14). Previous studies reported that the most frequent disturbances reported by MS patients included terminal insomnia, followed by middle and initial insomnias. Waking too early has been described as a common problem among MS patients (Stanton et al. 2006; Caminero and Bartolomé 2011).

Depression may also contribute to insomnia in MS as we figure out those patients who were using antidepressant medications have less insomnia than who did not. Identification and aggressive management of these problems are important, as there is mounting evidence of a bidirectional relationship between sleep and depression, such that insomnia treatment may improve symptoms of these other disorders (Taylor et al. 2007).

### Study limitations

This study had some methodological limitations. The inclusion of MS patients initially depended upon patients’ records and self-rating scales and did not include physician-dependent scales. The sample size was limited due to the strict inclusion criteria followed in this study. In future, a study with a larger sample may be planned to unravel this issue. The assessment of some associated symptoms, (e.g., fatigue or nocturia), which may constitute major causes for sleep disturbance among MS patients, were not assessed in this study. Body mass index of patients was not assessed in relation to insomnia. MS patients with insomnia were not further categorized into different types of insomnia. Moreover, the researchers considered that depressed MS patients, whether on antidepressant medications or not, should be studied, though antidepressant medications may affect their sleep. However, since the followed study design was cross-sectional, we could not examine the isolated effect of antidepressant therapy on either of the insomnia groups.

## Conclusions

This study demonstrated a low prevalence of insomnia among non-depressed MS patients. Insomnia was associated with higher educational level but no significant association with degree of disability, duration of disease, or MS treatment used. Additional research, particularly with larger sample sizes, is needed to assess further the level of insomnia for depressed and non-depressed MS patients in Saudi Arabia.

## Abbreviations

ISI: Insomnia Severity Index
MS: Multiple sclerosis
PDDS: Patient Determined Disease Steps
PHQ-9: Patient Health Questionnaire-9
